# Caries experience among children and adolescents from a longitudinal Swedish national registry study over a 10-year period

**DOI:** 10.2340/aos.v85.45204

**Published:** 2026-01-07

**Authors:** Håkan Flink, Anders Hedenbjörk-Lager, Simon Liljeström, Eva Nohlert, Åke Tegelberg

**Affiliations:** a Region Västmanland, Uppsala University, Centre for Clinical Research Västerås, Västerås Hospital, Västerås, Sweden; b Faculty of Odontology, Malmö University, Malmö, Sweden

**Keywords:** Caries experience, children and adolescents, disease progression, epidemiology, life-course perspective

## Abstract

**Objective:**

To identify caries experience in two groups of children and adolescents using longitudinal data from the Swedish Quality Registry for Caries and Periodontal Diseases (SKaPa).

**Material and methods:**

Data from two groups (10- and 20-year-olds), 165,365 individuals, were observed retrospectively for 10 years. Using a three-trajectory caries model (3-TCM), individuals were assigned according to their caries development as: high (15%), moderate (45%), or low (40%). Caries experience was expressed using the decayed and filled surfaces (DFS) and dfs indices. The specific affected caries (SaC) index and a point prevalence measurement at age 6 years (PP-6) were also analyzed.

**Results:**

Over the observation period, significant differences were discovered between all three trajectories within the oldest group (20-year-olds). The mean DFS increase was significantly elevated for the high trajectory compared with the lower trajectories, 7.9 ± 10.2, 3.3 ± 2.9 and 0.2 ± 0.5 for the three trajectories respectively. In the youngest group (10-year-olds), the high trajectory combined with the SaC and PP-6 provided further information of those with the highest caries experience.

**Conclusions:**

The 3-TCM identified individuals with high caries experience in the permanent dentition; but to properly elucidate caries experience in the primary and mixed dentitions, a combination with other indices was needed.

## Introduction

Untreated dental caries is the most prevalent non-communicable disease of both the primary and permanent dentitions globally [1, 2]. However, caries prevalence has decreased in many countries, and an increasing number of individuals now display few or no caries lesions. The most common method for describing the extent of caries experience is the decayed, missing, and filled (DMF) index [3, 4]. Despite the decreases reflected by the DMF index [5, 6], there is still a part of the population with recurrent caries lesions, hidden by the skewed distribution that needs to be examined [7, 8].

An earlier method to manage the skewed distribution problem in relation to the DMF index is the significant caries (SiC) index, which has been used on international World Health Organization (WHO) data regarding 12-year-olds [9]. A large group of individuals still displayed high or very high DMF values, despite most of the population being caries-free. Clearly, the mean DMF values did not reflect the skewed distribution accurately, thereby leading to a risk of incorrect conclusions regarding the caries situation for the whole population in this age group.

Another way to investigate the natural history of dental caries experience is the three-trajectory caries model (3-TCM) described by Broadbent and colleagues [10]. In this model, the population is assigned to one of the three DMFS (DMF surfaces in permanent teeth) index-based groups. The model has been useful for identification of the adults with the highest caries experience, which is also the group most likely to exhibit recurring caries lesions (15% of the population) [11, 12]. In the youngest group (aged 20 years), the DMFS index differed significantly among the three trajectories [11]. This implies that caries disease begins at a young age [13–15] and that the disease often continues into adulthood [14].

By calculating the DMF index for the third of the population with the highest caries experience, the SiC index was intended to provide a more accurate picture of the actual caries situation. However, continual increases in oral health and the resulting decline in mean caries experience have led to the development of the specific affected caries (SaC) index, which includes only children with caries experience [16].

Systematic reviews of caries prediction among children and adolescents have concluded that previous caries experience is a significant risk factor for further caries disease and that it is the most powerful single predictor variable in all age groups [17, 18]. Early caries experience in the primary dentition at younger ages increase the risk for new caries in childhood and adolescence [13]. This development also tends to progress in young adults [19, 20]. Early caries in the primary dentition may thus be particularly important to monitor [21].

Identification of the group of individuals with the highest caries experience may illuminate their prevention needs, and possibly aid in the development of more efficient preventive methods for this patient group [22]. Because the 3-TCM model can identify the adults with the highest caries experience over time, this study investigated whether a similar pattern can be discerned also in younger age groups. Combining this with the SaC index describes the proportion of individuals with caries experience more clearly [16]. The SaC index may be more important in the youngest group (10-year-olds) because these children had mainly primary teeth during the observation period and because the accuracy of the 3-TCM in the mixed dentition is unclear. Analyzing the SaC index along with a point prevalence of caries experience at age of 6 years (PP-6), which has been described as a caries prediction model in adolescents [13], may provide additional information about the caries experience in this age group.

The Swedish Quality Registry for Caries and Periodontal Disease (SKaPa) provides data from all age groups and offers a unique way to investigate the caries experience of the entire Swedish population [23], including young groups and both the primary and permanent dentitions.

The aim herein was thus to identify the caries experiences in child and adolescent groups from a national registry (SKaPa) over a 10-year period using the 3-TCM combined with the SaC index and PP-6.

We hypothesized that use of the 3-TCM, in combination with the SaC index and PP-6, can be additional in identifying the individuals with the highest caries experience in groups, including both the primary and permanent dentitions.

## Material and methods

**Figure 1:**
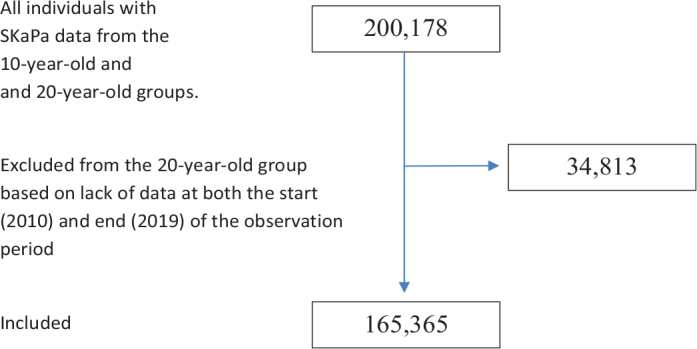
Flow chart of eligible sample (with SKaPa data), excluded (missing SKaPa data during 2010 and 2019) and included in analyses.

This retrospective study was conducted following the Strengthening Reporting Observational Studies in Epidemiology (STROBE) guidelines [24, 25]. Longitudinal caries data for two age groups, aged 10 and 20 years in 2019 was retrieved from the SKaPa registry for a period of 10 years. The included individuals were regular attendees at one of 1,182 general dental clinics in Sweden. A total of 165,365 individuals were included in the analyses (Figure 1). In these age groups, more than 80% have received dental examination (during a 3-year period) from the age of 3 years [26]. The examinations were performed by dentists or dental hygienists predominantly in public dental clinics. The dental service in 2019 was free of charge up to the age of 23 years in Sweden.

The main variables were the DFS (decayed and filled surfaces) index for the permanent dentition, with a range of 0–32 teeth and a maximum of 148 tooth surfaces, and the dfs (decayed and filled surfaces) index for the primary dentition, with a range of 0–20 teeth and a maximum of 88 tooth surfaces.

The group-based trajectory model, described in the Dunedin cohort study [10], is a specialized application of finite mixture modeling. This approach simplifies analyses of longitudinal data by identifying developmental trajectory groups on a likelihood basis. This involves approaching a set of individual trajectories by grouping those that closely resemble one another using a probability function. Dealing with fewer group trajectories is less complicated than analyzing several thousand individual trajectories [10, 27]. Herein, a similar model was applied by adapting the proportions from the Dunedin cohort analyses [10] to facilitate comparisons and evaluations of the outcomes from different populations.

We analyzed the mean DFS (decayed and filled surfaces in permanent teeth), instead of the full DMFS index, and dfs (decayed and filled surfaces in primary teeth) instead of the defs index, as the missing (M) and extracted (e) components are ambiguous in younger groups [28]. DFS index values for the permanent dentition in both age groups were assigned to three different trajectories at the end of the observation period in 2019: high, 15% of the population; moderate, 45%; and low, 40% (Table 1). The method has been described in previous studies of adult SKaPa data [11, 12].

**Table 1:** Sample sizes of 10- and 20-year-old SKaPa groups classified into three caries experience trajectories.

Age group, years*	High (15%)	Moderate (45%)	Low (40%)	Total
**10**	16,299	48,897	43,464	108,660
**20**	6,589	25,343	24,773	56,705
**Total**	22,888	74,240	68,237	165,365

* Age at end of observation period (2019).

In the 20-year-old group, the same model used in previous studies was applied [11, 12]. The mean DFS index values for all trajectories were calculated at the start (2010) and at the end (2019) of the observation period and used to quantify caries experience over time (Table 2). The mean increase in the DFS index during the observation period was used as the main variable to quantify the caries increment for each trajectory. The mean dfs index for the primary dentition was calculated in 2010 and 2019 according to the trajectory group classification of the permanent dentition in 2019.

**Table 2:** Mean DFS values at the end (2019) and start (2010) of the observation period for the three caries trajectories in the 10- and 20-year-old SKaPa groups.

	High (15%)		Moderate (45%)		Low (40%)		Total	
Age group, years*	*N*	Mean ± SD	*N*	Mean ± SD	*N*	Mean ± SD	*N*	Mean ± SD
**10**								
DFS 2019	16,299	1.1 ± 2.5	48,897	0.9 ± 1.4	43,464	0.00 ± 0.00	108,660	0.6 ± 1.4
**20**								
DFS 2010**	6,589	2.2 ± 3.4	25,343	1.2 ± 1.7	24,773	0.1 ± 0.3	56,705	0.8 ± 1.8
DFS 2019	6,589	10.1 ± 11.5	25,343	4.5 ± 3.2	24,773	0.3 ± 0.5	56,705	3.3 ± 5.5
Increase**		7.9 ± 10.2		3.3 ± 2.9		0.2 ± 0.5		2.5 ± 4.7

SD: standard deviation; DFS: decayed and filled surfaces (permanent dentition).

* Age at end of observation period (2019);

** only 20-year-old group.

In the 10-year-old group, mean DFS index values for all trajectories were calculated at the end of the observation period (2019). However, the caries experience in the permanent dentition could not be surveyed using the same methodology (Table 2). Instead, mean annual dfs and DFS indices were calculated for each trajectory as far back as possible in both the 10-year-old (Table 3a) and 20-year-old groups (Table 3b).

**Table 3a:** Mean annual dfs and DFS values for each caries trajectory group in the 10-year-old SKaPa group.

Age, years	1	2	3	4	5	6	7	8	9	10
	*N* = 7,621 Mean ± SD	*N* = 18,192 Mean ± SD	*N* = 74,091 Mean ± SD	*N* = 83,437 Mean ± SD	*N* = 91,866 Mean ± SD	*N* = 95,801 Mean ± SD	*N* = 99,754 Mean ± SD	*N* = 102,967 Mean ± SD	*N* = 105,841 Mean ± SD	*N* = 108,656 Mean ± SD
dfs high (15%)	0.1 ± 0.3	0.1 ± 1.4	0.2 ± 1.6	0.5 ± 2.3	0.9 ± 3.1	1.3 ± 3.6	1.7 ± 3.9	2.0 ± 4.1	2.1 ± 4.0	2.0 ± 3.7
dfs moderate (45%)	0.0 ± 0.6	0.1 ± 1.3	0.2 ± 1.5	0.5 ± 2.4	1.0 ± 3.2	1.5 ± 3.7	2.0 ± 4.1	2.3 ± 4.3	2.5 ± 4.2	2.3 ± 3.9
dfs low (40%)	0.0 ± 0.1	0.1 ± 0.8	0.1 ± 1.1	0.3 ± 1.7	0.6 ± 2.3	0.9 ± 2.8	1.2 ± 3.1	1.5 ± 3.3	1.6 ± 3.3	1.5 ± 3.1
dfs total	0.0 ± 0.5	0.1 ± 1.1	0.2 ± 1.4	0.4 ± 2.1	0.8 ± 2.8	1.2 ± 3.4	1.6 ± 3.7	1.9 ± 3.9	2.1 ± 3.9	1.9 ± 3.6
DFS high (15%)							0.3 ± 1.1	0.6 ± 1.6	0.9 ± 2.1	1.1 ± 2.5
DFS moderate (45%)							0.2 ± 0.7	0.4 ± 1.0	0.7 ± 1.2	0.9 ± 1.4
DFS low (40%)							0.0 ± 0.0	0.0 ± 0.0	0.0 ± 0.0	0.0 ± 0.0
DFS total							0.1 ± 0.6	0.3 ± 0.9	0.4 ± 1.2	0.6 ± 1.4

SD: standard deviation; dfs: decayed and filled surfaces (primary dentition); DFS: decayed and filled surfaces (permanent dentition).

**Table 3b:** Mean annual dfs and DFS values for each caries trajectory group in the 20-year-old SKaPa group of 56,705* individuals.

Age, years	11	12	13	14	15	16	17	18	19	20
	Mean ± SD	Mean ± SD	Mean ± SD	Mean ± SD	Mean ± SD	Mean ± SD	Mean ± SD	Mean ± SD	Mean ± SD	Mean ± SD
dfs high (15%)	2.3 ± 3.7	1.5 ± 3.0								
dfs moderate (45%)	1.8 ± 3.1	1.2 ± 2.5								
dfs low (40%)	0.9 ± 2.0	0.6 ± 1.7								
dfs total	1.5 ± 2.8	0.9 ± 2.2								
DFS high (15%)	2.2 ± 3.4	2.8 ± 4.1	3.6 ± 4.9	4.4 ± 5.7	5.4 ± 6.7	6.3 ± 7.8	7.4 ± 8.8	8.3 ± 9.7	9.3 ± 10.6	10.1 ± 11.5
DFS moderate (45%)	1.2 ± 1.7	1.5 ± 1.9	1.9 ± 2.2	2.2 ± 2.3	2.7 ± 2.5	3.0 ± 2.7	3.4 ± 2.9	3.8 ± 3.0	4.1 ± 3.1	4.5 ± 3.2
DFS low (40%)	0.1 ± 0.3	0.1 ± 0.3	0.1 ± 0.4	0.2 ± 0.4	0.2 ± 0.4	0.2 ± 0.4	0.2 ± 0.5	0.3 ± 0.5	0.3 ± 0.5	0.3 ± 0.5
DFS total	0.8 ± 1.8	1.0 ± 2.1	1.3 ± 2.6	1.7 ± 3.0	2.0 ± 3.6	2.4 ± 4.2	2.9 ± 4.8	3.2 ± 5.4	3.7 ± 6.0	4.1 ± 6.5

SD: standard deviation; dfs: decayed and filled surfaces (primary dentition); DFS: decayed and filled surfaces (permanent dentition).

* All individuals in the group were analyzed at each age. In the SKaPa registry, when data for a specific year were missing, the value from the previous year was carried forward.

Retrospective mean dfs, DFS, and SaC indices (SaC_dfs_ and SaC_DFS_, respectively), combined with information about the percentage of caries-free dentitions (dfs = 0 and DFS = 0, respectively) were calculated for each year of age in the 10-year-old (Table 4a) and 20-year-old (Table 4b) groups.

**Table 4a:** Annual percentages with caries-free dentitions (dfs = 0 and DFS = 0) and mean dfs and DFS values (SaCdfs and SaCDFS) in the 10-year-old SKaPa group.

Age, years	1	2	3	4	5	6	7	8	9	10
	*N* = 7,621 Mean ± SD	*N* = 18,192 Mean ± SD	*N* = 74,091 Mean ± SD	*N* = 83,437 Mean ± SD	*N* = 91,866 Mean ± SD	*N* = 95,801 Mean ± SD	*N* = 99,754 Mean ± SD	*N* = 102,967 Mean ± SD	*N* = 105,841 Mean ± SD	*N* = 108,656 Mean ± SD
dfs = 0 (%)	99%	99%	96%	91%	85%	79%	71%	66%	62%	62%
dfs (m ± SD)	0.0 ± 0.5	0.1 ± 1.1	0.2 ± 1.4	0.4 ± 2.1	0.8 ± 2.8	1.2 ± 3.4	1.6 ± 3.7	1.9 ± 3.9	2.1 ± 3.9	1.9 ± 3.6
SaC _dfs_	8.3 ± 6.3	6.4 ± 6.8	4.5 ± 5.1	4.9 ± 5.6	5.2 ± 5.5	5.6 ± 5.3	5.7 ± 5.1	5.7 ± 4.8	5.5 ± 4.5	5.1 ± 4.3
dfs > 0	*N* = 15	*N* = 252	*N* = 3,115	*N* = 7,214	*N* = 14,093	*N* = 20,526	*N* = 28,665	*N* = 34,985	*N* = 40,398	*N* = 41,029
DFS = 0							93%	87%	81%	77%
DFS (m ± SD)							0.1 ± 0.6	0.3 ± 0.9	0.4 ± 1.2	0.6 ± 1.4
SaC _DFS_ DFS > 0							1.9 ± 1.4 *N* = 6,957	2.2 ± 1.6 *N* = 13,324	2.3 ± 1.8 *N* = 20,232	2.5 ± 2.0 *N* = 25,341

SD: standard deviation; dfs: decayed and filled surfaces (primary dentition); DFS: decayed and filled surfaces (permanent dentition).

**Table 4b:** Annual percentages of caries-free dentitions (dfs = 0 and DFS = 0) and mean dfs and DFS values (SaCdfs and SaCDFS) in the 20-year-old SKaPa group of 56,705* individuals.

Age, years	11	12	13	14	15	16	17	18	19	20
	Mean ± SD	Mean ± SD	Mean ± SD	Mean ± SD	Mean ± SD	Mean ± SD	Mean ± SD	Mean ± SD	Mean ± SD	Mean ± SD
dfs = 0 (%)	64%	73%								
dfs (m ± SD)	1.5 ± 2.8	0.9 ± 2.2								
SaC _dfs_	4.1 ± 3.4	3.6 ± 3.1								
dfs > 0	*N* = 19,630	*N* = 14,826								
DFS = 0	69%	64%	59%	55%	50%	47%	44%	42%	40%	38%
DFS (m ± SD)	0.8 ± 1.8	1.0 ± 2.1	1.3 ± 2.6	1.7 ± 3.0	2.0 ± 3.6	2.4 ± 4.2	2.9 ± 4.8	3.2 ± 5.4	3.7 ± 6.0	4.1 ± 6.5
SaC _DFS_ DFS > 0	2.7 ± 2.4 *N* = 17,688	2.9 ± 2.7 *N* = 20,414	3.2 ± 3.0 *N* = 23,432	3.5 ± 3.4 *N* = 25,624	3.8 ± 3.9 *N* = 28,130	4.1 ± 4.4 *N* = 29,809	4.5 ± 4.9 *N* = 31,633	4.8 ± 5.3 *N* = 32,861	5.0 ± 5.7 *N* = 34,278	5.3 ± 6.1 *N* = 35,192

SD: standard deviation; dfs: decayed and filled surfaces (primary dentition); DFS: decayed and filled surfaces (permanent dentition).

* All individuals in the group were analyzed at each age. In the SKaPa registry, when data for a specific year were missing, the value from the previous year was carried forward.

To further evaluate the value of the SaC_dfs_ index, included individuals were divided into three groups based on their caries experience at the age of 6 years (i.e. PP-6) [13]. First, those with no caries (dfs = 0) and those with caries (dfs > 0) were separated. Then, the group with caries (dfs > 0) was divided again into two equally sized groups based on the degree of caries experience, dfs and DFS development over time were analyzed in these three groups at 6–10 years (during 2016–2019) for both primary and permanent teeth (Figure 6). The Swedish Ethical Review Authority approved the research project (Dnr 2022-01689-02).

### Statistical analysis

Descriptive statistics were used to characterize caries development throughout the observation period. Mean values, including mean differences, were compared within groups using dependent-samples t tests. To assess differences between trajectories and ages, mixed-effects ANOVAs were applied. Because of the large sample sizes, a conservative significance level was adopted, with *p*-values .01 considered statistically significant.

To aid interpretation of the 20-year-old group results, we also calculated effect sizes, a standardized measure of the strength of the relations between variables. When two variables were compared, Cohen’s d [29] was calculated by dividing the mean difference by the pooled standard deviation. Effect sizes were interpreted using Cohen’s guidelines: 0.2 represents a small effect, 0.5 a moderate effect, and 0.8 a large effect.

Data were analyzed using IBM SPSS Statistics version 28 for Windows (IBM Corporation; Armonk, NY, USA).

## Results

The sample sizes in each age group and trajectory group are shown in Table 1. The registry data from 2019 included 200,178 individuals: 108,660 in the 10-year-old group (87% of the 124,897 individuals in the entire Swedish population in the age group) and 91,518 in the 20-year-old group (78% of the 116,615 individuals in the entire Swedish population in the age group) [30].

Among the 20-year-old group, 34,813 had not received a dental examination in 2010 and were therefore excluded (Figure 1). This exclusion was done to assure longitudinal data from the same individuals over a 10-year period (as described in previous studies among adults using the same method) [11, 12], resulting in 56,705 individuals remaining in the analysis (48% of the entire national age group).

**Figure 2:**
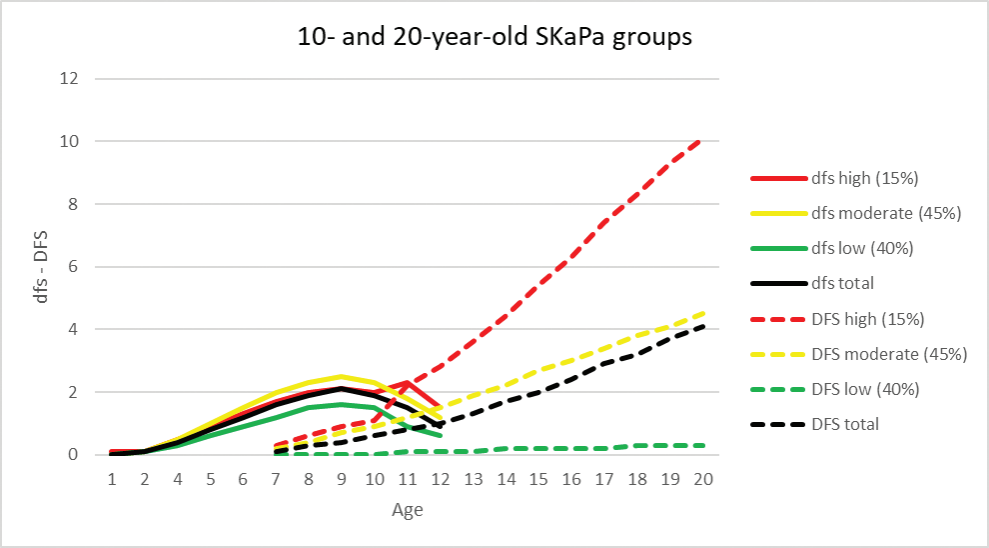
Progression of the dfs and DFS indices in the primary and permanent dentitions for the high, moderate, and low trajectories, and total mean values in the 10- and 20-year-old groups during the 2010–2019 observation period (dfs/DFS = decayed and filled tooth surfaces).

The mean DFS index increase in the 20-year-old group during the 10-year observation period was significant for all three trajectories (Tables 2, 3b and Figure 2). The DFS index increase was significantly higher in the high-trajectory group compared to the low- and moderate-trajectory groups. Effect sizes were larger in the moderate- and high-trajectory groups than in the low-trajectory group, reflecting a more pronounced DFS index increase for the moderate- and high-trajectory groups. These effect sizes were moderate and large [29]. In this group, the mean DFS increase $\left(\bar{d}\pm sd\right)$ and Cohen’s *d* effect sizes between start (2010) and end (2019) of the observation period were 8.0 ± 10.2, with *d* = 0.8 for the high-trajectory, 3.2 ± 2.9, with *d* = 1.1 for the moderate-trajectory, and 0.2 ± 0.5, with *d* = 0.5 for the low-trajectory. All effect size differences between trajectories were statistically significant (*p* < 0.001).

**Figure 3:**
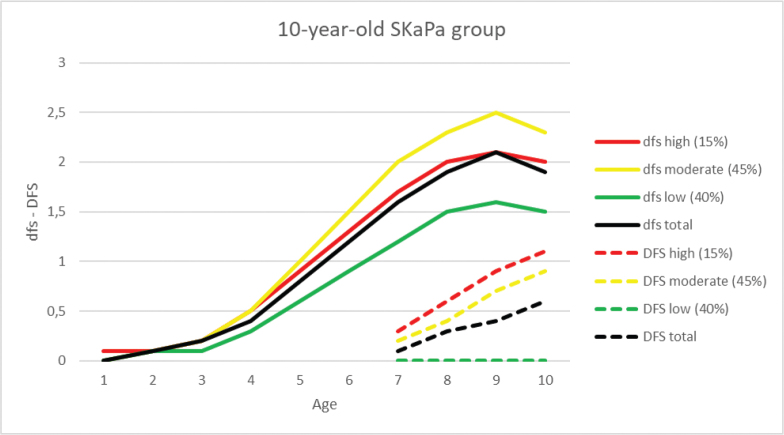
Progression of the dfs and DFS indices in the primary and permanent dentitions for the high, moderate, and low trajectories, and total mean values in the 10-year-old age group during the 2010–2019 observation period (dfs/DFS = decayed and filled tooth surfaces).

In the 10-year-old group, DFS index values were monitored retrospectively until the age of 6 years. The expressed caries development pattern in the permanent dentition was similar to the 20-year-old group, except in the low-trajectory group, which had a value of 0 at both 6 and 10 years of age (Table 3a, Figures 2 and 3).

**Figure 4:**
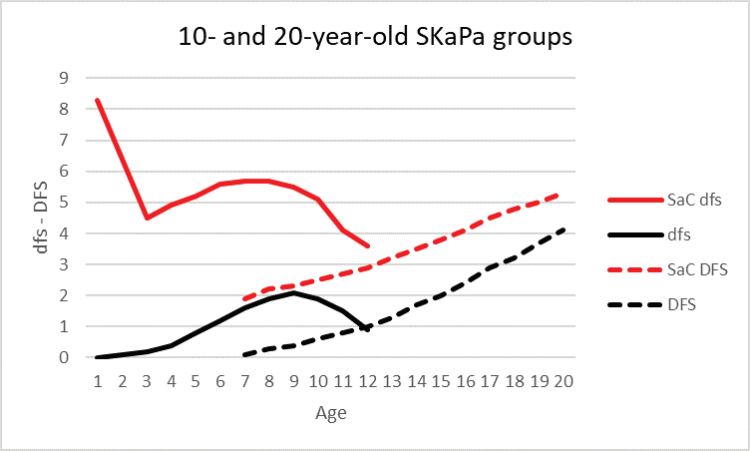
Progression of the SaC index in children and adolescents with caries experience (dfs/DFS > 0); expressed as SaCdfs (primary dentition), along with dfs, SaCDFS (permanent dentition), and DFS index annually from age 1–20 years during the 2010–2019 observation period (dfs/DFS = decayed and filled tooth surfaces).

**Figure 5:**
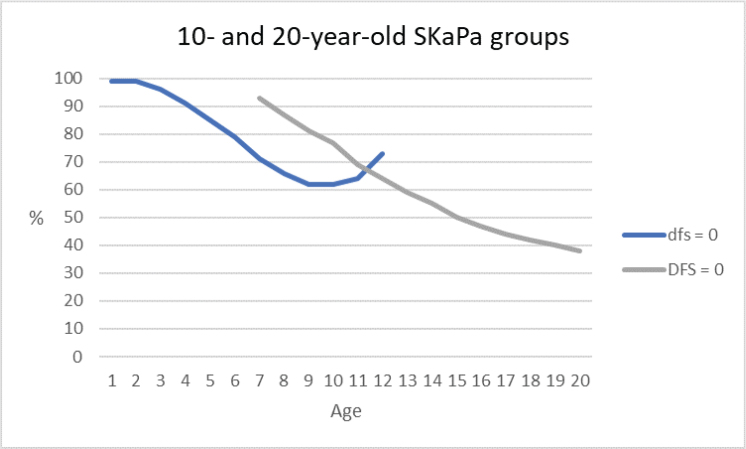
Annual prevalence of caries-free individuals according to both dfs (primary dentition) and DFS (permanent dentition) during the 2010–2019 observation period.

In the primary dentition, the dfs index for each of the three trajectories and total mean values for the 10-year-old group during the observation period are shown in Table 3a and Figures 2 and 3. The dfs increase with a peak at age 9 years was observed for all trajectories. When comparing caries progression throughout the observation period, the SaC index was higher than the dfs and DFS indices at all ages (1–20 years, Figure 4). The difference was most pronounced for SaC_dfs_ compared with dfs in 1–3-year-olds, among whom >96% were caries-free (Figure 5). Further, >60% of both groups had no experience of caries in the primary dentition during the observation period (Figure 5).

**Figure 6:**
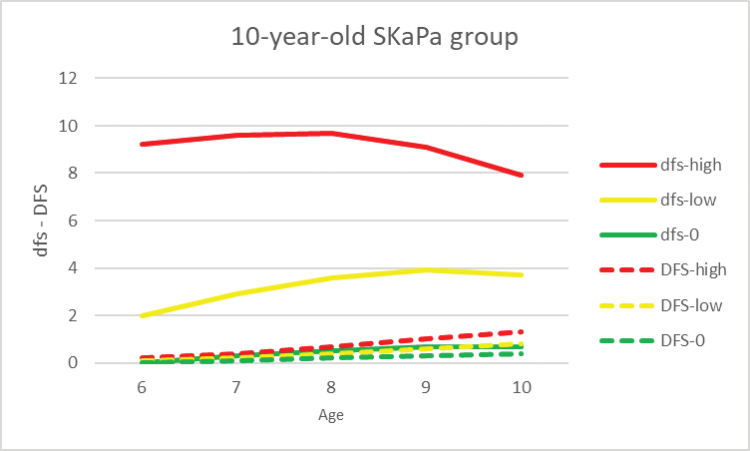
Caries development by caries experience at age 6 years, in those with no caries (dfs = 0, ≈80%) and those with caries (dfs > 0), divided into dfs low (≈10%) and dfs high (≈10%) groups; annual caries experience in both dfs (primary dentition) and DFS (permanent dentition) at ages 6–10 years (during 2016–2019) (dfs/DFS = decayed and filled tooth surfaces).

To investigate the 10-year-old group regarding caries experience (dfs), the sample was divided into three groups, as described in the method section (i.e. no caries ≈ 80%, low ≈ 10%, high ≈ 10%) (Figure 6). Thus, mixed-effects ANOVAs were calculated for each of the primary and permanent dentitions. For the primary dentition, there were main effects for group (no caries, low, or high) (*F*_2, 95,798_ = 88,331, *p* < 0.001) and age (*F*_4, 383,192_ = 3,077, *p* < 0.001), as well as an interaction effect (*F*_8, 383,192_ = 2,719, *p* < 0.001). Likewise, for the permanent dentition, there were main effects for group (*F*_2, 95,788_ = 2,519, *p* < 0.001) and age (*F*_4, 383,152_ = 10,052, *p* < 0.001), as well as an interaction effect (*F*_8, 383,152_ = 1,177, *p* < 0.001). For both the primary and permanent dentitions, the interactions indicate different caries development patterns over time for different groups (Figure 6). Moreover, it is noteworthy that caries development as described by dfs declined from age 8–9 years in the high group, whereas there was a progressive increase for all three DFS groups.

## Discussion

Caries experience in both age groups was monitored over the observation period. The group with the highest caries experience demonstrated an elevated rate of recurrent caries lesions. Previous studies on SKaPa registry data for adults using the 3-TCM [11, 12] found similar results regarding caries experience hidden by skewed distribution.

The aim herein was to use the 3-TCM in combination with the SaC index and the PP-6 to analyze data from a nationwide sample. The latter two indices may provide extended information about the proportion of caries-free individuals and specific mean dfs, as well as DFS values in the caries-active portion of the population. This was especially valuable in the youngest group (10-year-old), where hidden caries in the primary dentition may be useful to predict future caries development in the permanent dentition.

The study was not without limitations. First, the use of the 3-TCM with trajectory proportions based on the Dunedin study results may have been influenced by variations in caries prevalence in different populations. Further analysis concerning alternative trajectory proportions is thus needed. Second, the accuracy of the deft/DMFT indices is significantly inferior to that of the dft/DFT indices [28], which motivated the use of the dfs/DFS indices. Uncertainty regarding the extracted/missing (e/M) components of these indices may cause difficulties with the registration of exfoliating primary teeth in the mixed dentition [15, 28] and present unclear reasons for permanent tooth extractions [28, 31]. Third, replacement of restorations in caries-active individuals is a challenge for correct clinical caries registration, especially in the primary dentition where >50% of restorations have been reported as replaced [13]. This is a possible reason for the shift between the high and moderate dfs trajectories, potentially related to a higher proportion of secondary caries in the high-trajectory group (i.e. not elevating the dfs index) and a higher proportion of extractions (i.e. lowering the dfs index) (Figure 3). After the age of 13 years, most primary teeth have or are about to exfoliate and thus have less clinical relevance. Furthermore, the eruption patterns in the permanent dentition in the 10-year-old group are variable, and therefore registrations might be less accurate.

### Strengths and weaknesses

The registry data at inclusion 2019 presented for the 10- and the 20-year-old groups were 87% and 78% respectively, of the entire Swedish population, which suggests a high reliability regarding the disease prevalence data.

A general possibility for selection bias that varies between the two groups might be that parents in Sweden are obliged to bring their child to dental examination, otherwise risking being reported to social security, while at the age of 18, the children become independent adults according to the law and therefore may choose not to attend dental examination. This fact may explain the difference in completeness between the two groups.

We do not obtain any special knowledge about the characteristics of the individuals included in the register or the ones that are excluded. In other studies in these age groups, avoidance from dental treatment has been related to more invasive treatment and dental fear [32], and also dental fear related to the amount of caries experience [33]. These aspects might be reasons for not attending dental examinations and participating in the present study and need more attention.

Notwithstanding these limitations, a strength of this study is that the SKaPa registry data have been validated, showing satisfactory reliability and accuracy regarding dental caries in 6- and 12-year-olds, and the SKaPa has been confirmed as a reliable source for registry-based research [28, 34]. Altogether, the value of the study must be considered high, mainly related to the large sample size of participants and the long observation time. The results herein confirm that the 3-TCM is also useful for evaluating the permanent dentition in child and adolescent populations, consistent with the findings by Hall–Scullin and colleagues, who concluded that ‘caries-free and caries-active children should be considered as two separate populations, and suggesting that different prevention strategies are required to address their different risk profiles [15]. The trajectory model has recently been used in a similar way to the present study, showing continuing caries development in 3–18% of the participants over an 8 year period, from 7 to 15 years of age [35].

It is well known that caries now is limited to relatively circumscribed groups which are characterized by low socioeconomic status (SES), migration background and language problems which needs special attention [36]. Information about SES has been investigated in different regions of Sweden, but was not available for the present study.

The evidence for successful caries prevention opportunities in younger age groups is limited leading to calls for more clinical pediatric dentistry research [37]. A high-risk approach may be the most reasonable in an era of declining caries, but there is a lack of evidence on how to prevent caries among the most caries-active 10% of the child population [38].

When investigating child caries prevalence from a life-course perspective [39, 40], it is necessary but challenging to start with early childhood caries (ECC), defined as the presence of one or more decayed or filled tooth surfaces in a child aged 6 years or younger [41].

Among those aged 1–3 years, the group with no caries experience (i.e. dfs = 0) predominated (Figure 5). The skewed distribution was identified by the SaC_dfs_ index, but remained hidden in the dfs index, which consequently underestimated caries in the primary dentition of that group, as well as in the older ages of the 10-year-old group (Figure 4). The SaC_dfs_ index described differences in caries experience more clearly than the trajectory model for that specific group. These findings suggest the need for improved caries prevention and special care for the highest trajectory groups in both age groups. Future research using longitudinal design is needed to clarify whether our results in a child population remain consistent in adolescents and later in life [20]. There is also a need to address intervention in pregnant women, new mothers, and other primary caregivers for preventing ECC [42], along with oral health birth cohort studies [43, 44]. New management protocols are needed, especially for individuals who remain in the high-trajectory groups of children and adolescents, including decisions about preventive care plans [45]. More systematic and precise descriptions of prevention method choices are needed in evaluations of their effects [46].

Data codes for different caries preventive treatments in the Swedish national dental system are difficult to analyze for several reasons. Many of the current codes are not disease-specific for caries, making evaluation of the effects of caries prevention almost impossible. There is also a need for differentiation of registry codes regarding in-clinic and homecare prevention recommendations, clearly specifying content and extent. Addressing these issues will be important for evaluation of the effects of different prevention methods using big datasets like SKaPa [47]. Detailed descriptions of preventive methods are also necessary to ensure that all dental care professionals perform them consistently [46, 48]. These future needs may confirm or refute if preventive methods work in real-world settings [49]. The recurrent caries lesions described in the present study may otherwise jeopardize interest and engagement in caries prevention among general dentists [50]. The possibilities to adapt and develop the Swedish code system must be considered as promising, making evaluation of caries preventive effects on longitudinal caries data of high trajectory individuals, a reality in real world settings.

In conclusion, the three-trajectory model was useful for the identification of high caries experience in the permanent dentition; but to elucidate the caries experience in the primary and mixed dentitions, a combination with other indices was needed.

## Data Availability

Data are available upon reasonable request to the corresponding author.
